# Heating of Large Endovascular Stents and Stent Grafts in Magnetic Particle Imaging—Influence of Measurement Parameters and Isocenter Distance

**DOI:** 10.1007/s00270-022-03324-7

**Published:** 2022-12-13

**Authors:** Franz Wegner, Anselm von Gladiss, Huimin Wei, André Behrends, Ulrike Grzyska, Malte M. Sieren, Julian Haegele, Matthias Graeser, Thorsten M. Buzug, Joerg Barkhausen, Thomas Friedrich

**Affiliations:** 1 Department of Radiology and Nuclear Medicine, University of Lübeck, Ratzeburger Allee 160, 23562 Lübeck, Germany; 2 Institute for Computational Visualistics, University of Koblenz-Landau, Universitätsstraße 1, 56070 Koblenz, Germany; 3 Institute of Medical Engineering, University of Lübeck, Ratzeburger Allee 160, 23562 Lübeck, Germany; 4 Fraunhofer Research Institution for Individualized and Cell-Based Medical Engineering IMTE, Mönkhofer Weg 239a, 23562 Lübeck, Germany; 5 Zentrum für Radiologie und Nuklearmedizin, Von-Werth-Straße 5, 41515 Grevenbroich, Germany

**Keywords:** Magnetic particle imaging, Stents, Heating, Safety

## Abstract

**Purpose:**

Magnetic particle imaging (MPI) is a tomographic imaging modality with the potential for cardiovascular applications. In this context, the extent to which stents are heated should be estimated from safety perspective. Furthermore, the influence of the measurement parameters and stent distance to the isocenter of the MPI scanner on stent heating were evaluated.

**Materials and Methods:**

Nine different endovascular stents and stent grafts were tested in polyvinyl-chloride tubes. The stents had diameters from 10 to 31 mm, lengths between 25 and 100 mm and were made from stainless steel, nitinol or cobalt-chromium. The temperature differences were recorded with fiber-optic thermometers. All measurements were performed in a preclinical commercial MPI scanner. The measurement parameters were varied (drive field strengths: 3, 6, 9, 12 mT and selection field gradients: 0, 1.25 and 2.5 T/m). Furthermore, measurements with different distances to the scanner’s isocenter were performed (100 to 0 mm).

**Results:**

All stents showed heating (maximum 53.1 K, minimum 4.6 K). The stent diameter directly correlated with the temperature increase. The drive field strength influenced the heating of the stents, whereas the selection field gradient had no detectable impact. The heating of the stents decreased with increasing distance from the scanner’s isocenter and thus correlated with the loss of the scanner’s magnetic field.

**Conclusion:**

Stents can cause potentially harmful heating in MPI. In addition to the stent diameter and design, the drive field strength and the distance to the MPI scanner’s isocenter must be kept in mind as influencing parameters.

## Introduction

Magnetic particle imaging (MPI) is an emerging tomographic imaging modality which visualizes the spatial distribution of superparamagnetic iron oxide nanoparticles (SPIONs) [1]. Over the last decade, numerous preclinical studies have proven MPI’s potential for cardiovascular imaging and interventional monitoring [2–9]. Here, the combination of real-time imaging capabilities and the lack of ionizing radiation renders MPI to be a promising imaging method. In addition, MPI has been proven to be especially advantageous for implantation and follow-up examination of endovascular stents [7, 10–12]. The stent lumen and stenoses can be quantified very accurately without the influence of material-induced artifacts. The latter strictly limit the established modalities CT and MRI concerning stent lumen evaluation [13, 14].

MPI is based on the application of static and oscillating magnetic fields. Therefore, heating of metallic objects due to eddy currents or magnetic hysteresis becomes an important safety issue. In the last years, first studies investigated the heating of interventional devices like metallic stents in MPI [15–17]. The stent diameter and design were identified as influencing parameters of stent heating.

In recent years, significant progress has been achieved in the development of clinical MPI scanners. Recently, the first human size MPI scanner was introduced for brain imaging [18]. This scanner offers a field of view (FOV) which could allow for the examination of human extremities. Thus, it is only a small step until the use of MPI for more clinical applications is technically feasible. Naturally, patients who require vascular imaging often have metallic endovascular stents. Therefore, it is of importance to investigate the potential extent of stent heating and its influencing parameters.

The purpose of this study was to determine the amount of stent heating in MPI by investigating stents with large diameters. The influence of the selection field gradient and the drive field strength of the MPI system was also studied to make the results comparable to other magnetic field combinations. Furthermore, to describe the magnetic fields’ influence on metallic objects outside the FOV, the heating in relation to the isocenter distance in the MPI scanner was analyzed in this work.

## Materials and Methods

### Stents and Vessel Phantoms

**Fig. 1:**
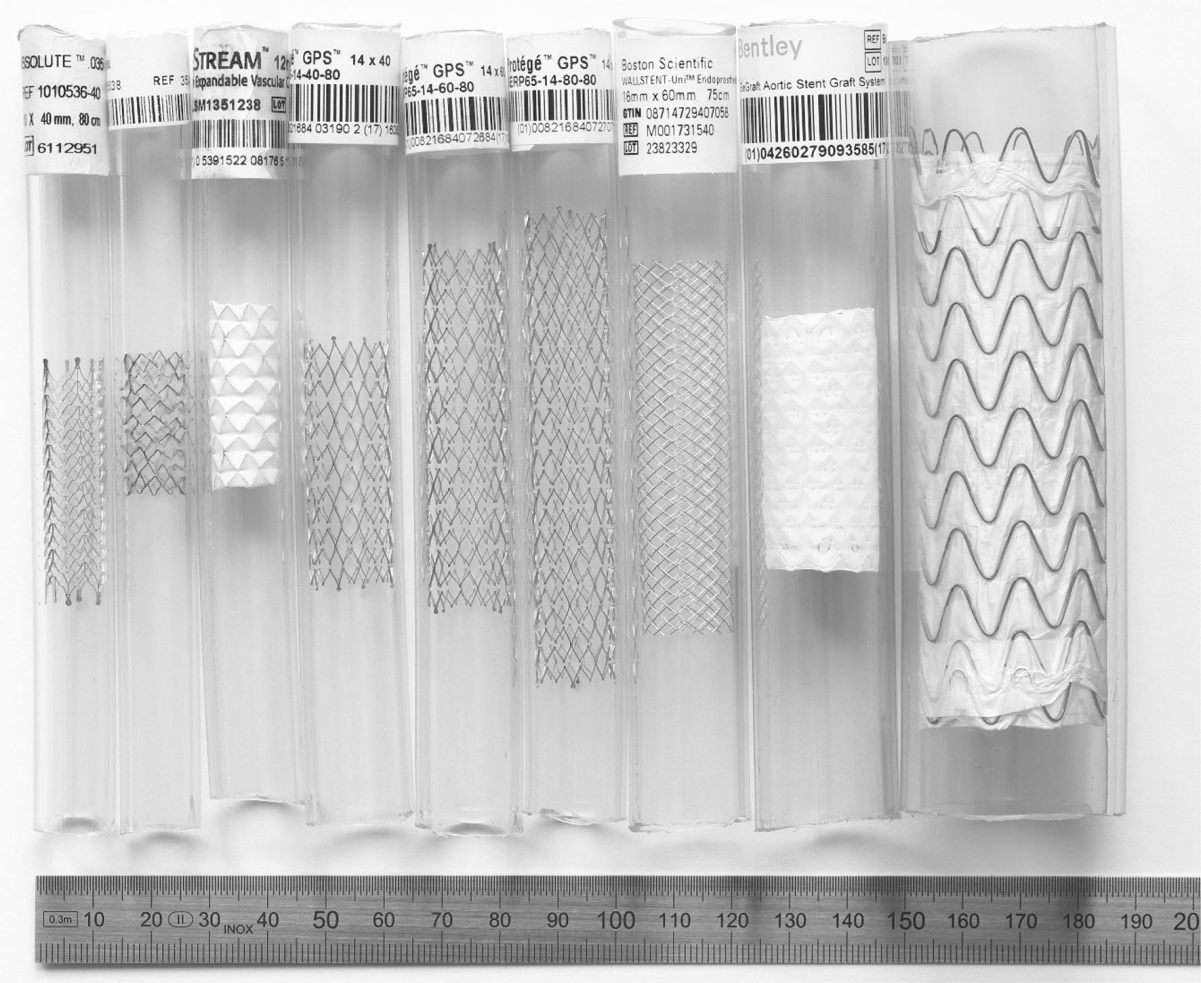
Overview of the stents and stent grafts in vessel phantoms. The order of the stents (left to right) is identical with Table 1 (downward)

**Fig. 2:**
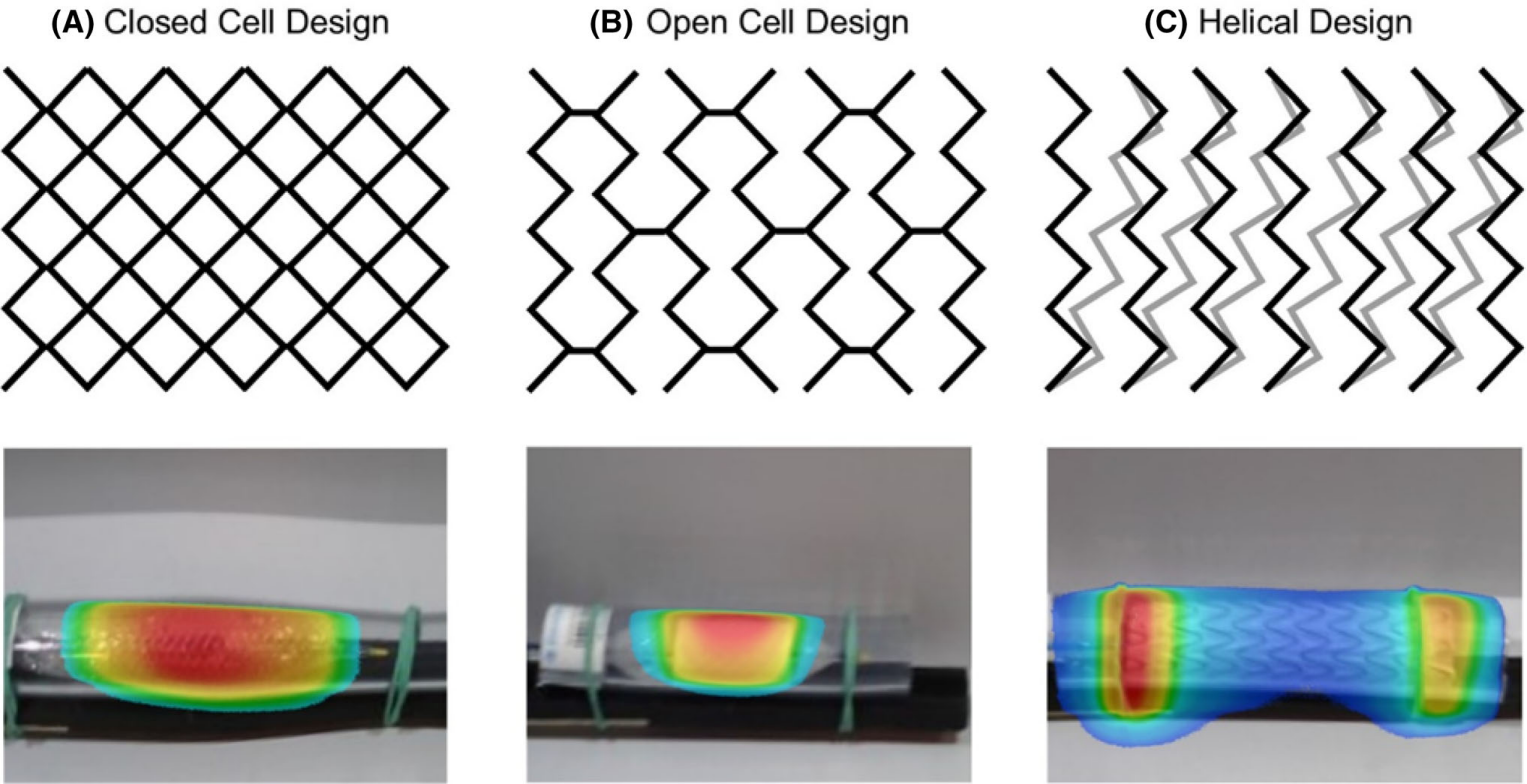
Schematic drawing of the three different stent designs and the exemplary thermographies which are showing the heat distribution after 431 s MPI scans. Since the period between the end of MPI scans and thermographies was not standardized, the temperatures are encoded semiquantitatively (blue is the coolest and red the warmest)

Nine different metallic endovascular stents and stent grafts (referred to in the following text as ‘stents’) were analyzed (Fig. 1, Table 1). The stents had diameters from 10 to 31 mm, lengths between 25 and 100 mm and were made from stainless steel, nitinol or cobalt-chromium. The stents can be assigned to three different design groups: closed cell, open cell and helical design (Fig. 2). All stents were implanted in polyvinyl-chloride vessel phantoms according to the nominal stent diameter with one exclusion—the Gore/TAG stent graft with a diameter of 31 mm was implanted in a phantom with a diameter of 30 mm.

**Table 1:** Stent details

Device type	Name	Ø (mm)	Length (mm)	Material	Design
Stent	Guidant/Absolute	10	40	Nitinol	Open cell
Stent	Biotronik/Dynamik	10	25	Stainless steel	Open cell
Stent graft	BARD/Lifestream	12	38	Stainless steel	Open cell
Stent	EV3/Protege GPS	14	40	Nitinol	Open cell
Stent	EV3/Protege GPS	14	60	Nitinol	Open cell
Stent	EV3/Protege GPS	14	80	Nitinol	Open cell
Stent	Boston scientific/Wallstent-Uni Endoprothesis	16	60	Nitinol	Closed cell
Stent graft	Bentley/BeGraft	20	48	Co-Cr	Open cell
Stent graft	Gore/TAG	31	100	Nitinol	Helical

### Temperature Measurements

The temperature measurements during the MPI scans were performed in accordance to already published measurement protocols [16, 17] with a fiber-optic thermometer (FTX-300-LUX + , Osensa, Coquitlam, Canada). As the stent design is expected to influence the heat distribution, an exemplary thermography (Testo 890–2, Testo, Lenzkirch, Germany) of one stent of each of the three different stent designs was performed a single time before the heating measurements to guarantee the temperature data acquisition to be conducted at the stents’ hot spots. For the closed cell stent design, the Boston Scientific/Wallstent-Uni Endoprothesis, for the open cell design the Bentley/BeGraft, and for the helical stent design, the Gore/TAG stent graft was imaged after an MPI scan of 430 s. This timeframe was chosen based on published measurement protocols [16, 17]. In accordance with the thermography results (see Fig. 2), the temperature probe was placed at the inner bottom of the stents’ center and directly fixed at the stent struts with air-filled balloon catheters. Only for the Gore/TAG stent graft, the temperature probe was additionally placed at the stent’s end because the thermography showed a different temperature distribution. For the measurements with varying stent positions in the scanner, two temperature probes were placed at the stent (center and end). As reference, a thermometer probe was placed at the bottom of the nonmagnetic phantom holder and fixed with tape for all measurements. The measured temperature curves were recorded continuously with the Osensa View FTX Professional software (Osensa, Coquitlam, Canada). The temperature differences were computed by subtracting the temperature after and before the MPI scans of each measurement. The reference temperatures were subtracted from the stents’ temperatures. Based on the inaccuracy of the measurement setup, a temperature increase in more than 0.1 K was defined as heating in this work.

### MPI Parameters

The temperature measurements were performed in a commercial preclinical MPI system (Bruker-Biospin, Ettlingen, Germany). The measurement parameters for the general heating measurements of the stents were the following: selection field gradients of 1.25 T/m in x- and y-direction and 2.5 T/m in z-direction. The drive field strength was 12 mT in each direction and the drive field frequencies were 24.510 kHz, 26.042 kHz, and 25.252 kHz in x-, y-, and z-direction, respectively.

To investigate the influence of different scan parameters, a variation in the drive field strength and the selection field gradient was performed. Therefore, drive field strengths of 3, 6, 9 and 12 mT in all directions and selection field gradients of 0, 1.25 and 2.5 T/m in z-direction, 0, 0.625 and 1.25 T/m in x- and y-direction were applied in the combinations shown in Table 2. Due to the comparable time dependent heating characteristic of stents in MPI [16, 17], we performed the experiments with a single stent model (Bentley/BeGraft) for a duration of 20,000 frames (431 s) and assume the results to be transferable to other stent types. This stent model has been selected, because it showed a significant heating and represents a common stent design. Additionally, the ramp up time of the drive field (~ 21 s) must be acknowledged.

**Table 2:** Temperature difference of the Bentley/BeGraft after 431 s MPI scan under the influence of different drive field strengths and selection field gradients

Drive field strength (mT)	Selection field gradient (T/m, in z-direction)	ΔT (K)
3	2.5	0
6	2.5	5.8
9	2.5	16.3
12	2.5	28.0
12	1.25	27.9
12	0	27.9

To evaluate the influence of the stent location in relation to the scanner’s isocenter on stent heating, the stent position varied. Exemplarily, the Boston Scientific/Wallstent-Uni Endoprothesis was placed on the phantom holder in 21 different positions along the x-axis of the scanner (from 100 to 0 mm distance from the FOV’s isocenter in steps of 5 mm). This stent model was chosen as it showed a homogenous heat distribution in the thermography (Fig. 2) and thus should be representative for stent heating in general. At each position, an MPI scan of 2,800 frames (60 s) was performed and the temperatures were recorded. To correlate the temperature increase to the drop in magnetic field when moving along the x-axis, a self-built pickup coil (66 mm diameter, x-direction) was used to determine the corresponding field profile in 1 mm steps between 0 and 100 mm from the isocenter. Therefore, drive field strengths of 10 mT in x-direction and 0 mT in y- and z-direction and selection field gradients of 2.5 T/m in x-direction and 0 T/m in y- and z-direction were applied.

### Data Analysis and Statistics

The temperature and voltage data were exported into MATLAB (Mathworks, Natick MA, USA), and the magnetic flux density was calculated and plotted. The correlation of stent diameter vs. heating and drive field strength vs. heating was computed by using the Pearson’s correlation coefficient.

## Results

### Thermography of Different Stent Designs

The thermographic images of the three different stent designs revealed different temperature distribution patterns with a homogenous distribution for the closed and open cell design (Fig. 2). In contrast, the helical stent design caused heating maxima at the stent’s ends.

### Heating During MPI Scans

**Fig. 3:**
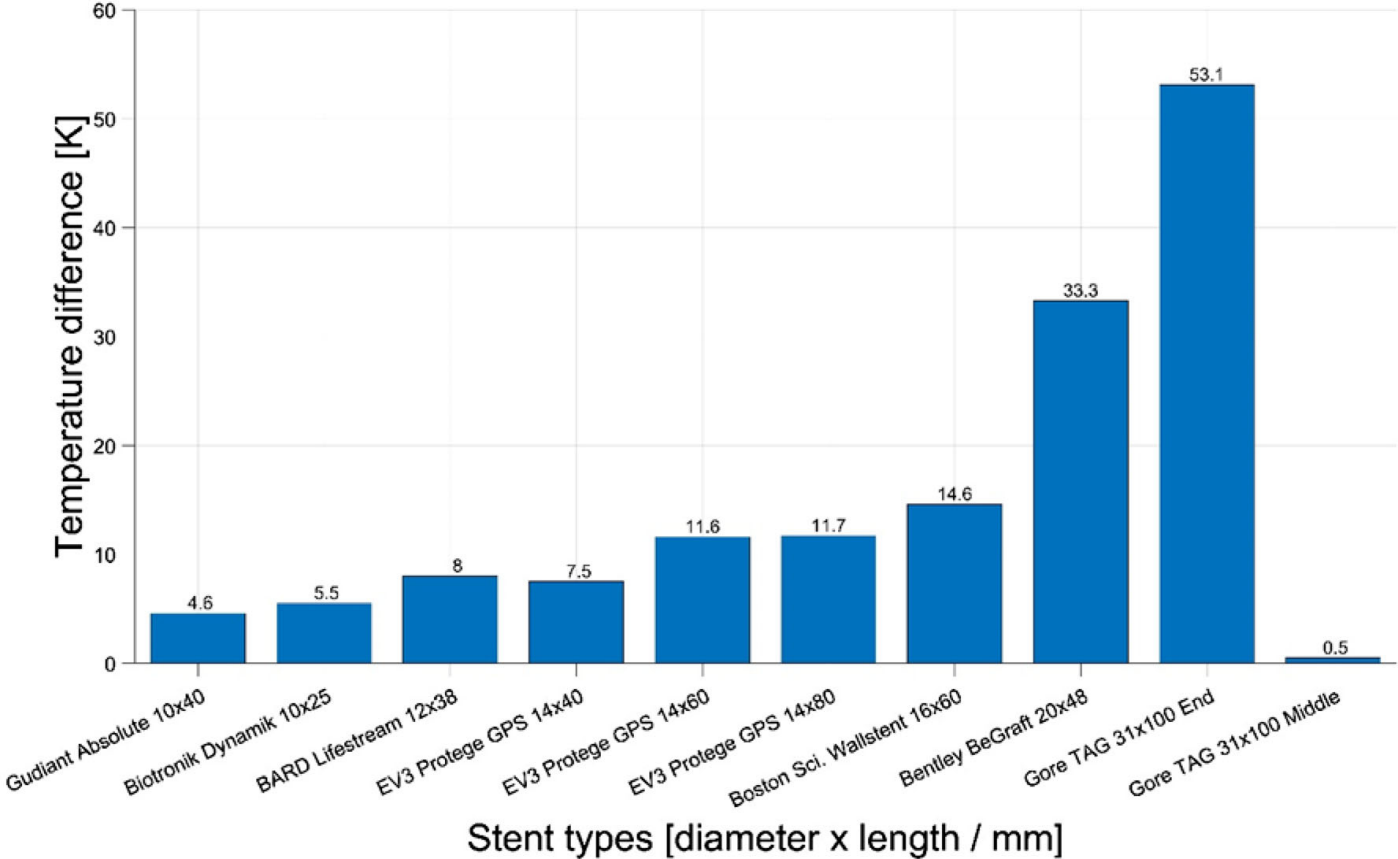
Temperature differences of the stents after 431 s MPI scans

During the MPI scans with a duration of 431 s, all stents showed a detectable temperature increase (Fig. 3). The minimum increase was 4.6 K (Guidant/Absolute) and the maximum was 53.1 K (Gore/TAG). The Gore/TAG stent graft showed only a slight heating of 0.5 K, when measured at the stent’s middle section, but the highest increase of 53.1 K at the stent’s ends (Fig. 2, 3). The measured temperature differences were rising with increasing diameter. Pearson’s correlation coefficient between square root of temperature difference and stent diameter depicted this relation with a value of *R* = 0.97 (with the exception of Gore/TAG in the middle section.).

### Variation in the Measurement Parameters

**Fig. 4:**
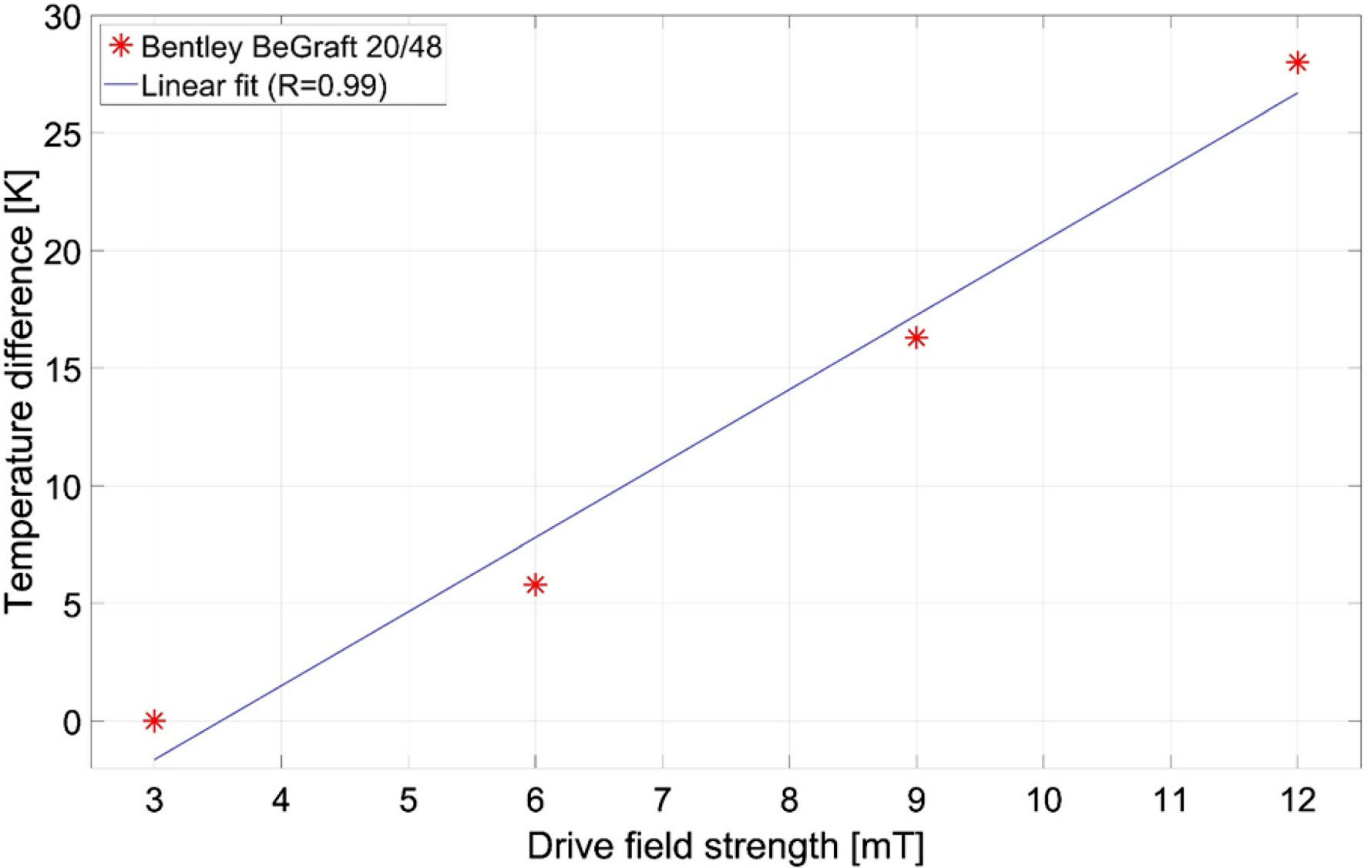
Linear fit of the temperature differences after scanning the Bentley/BeGraft for 431 s with different drive field strengths

The reduction in the drive field strengths resulted in a continuous decrease in the measured temperature differences (*R* = 0.99) (Fig. 4, Table 2). A variation in the selection field gradient did not show a relevant change of the resulting temperature increase.

### Variation in the Stent Distance to the Scanner’s Isocenter

**Fig. 5:**
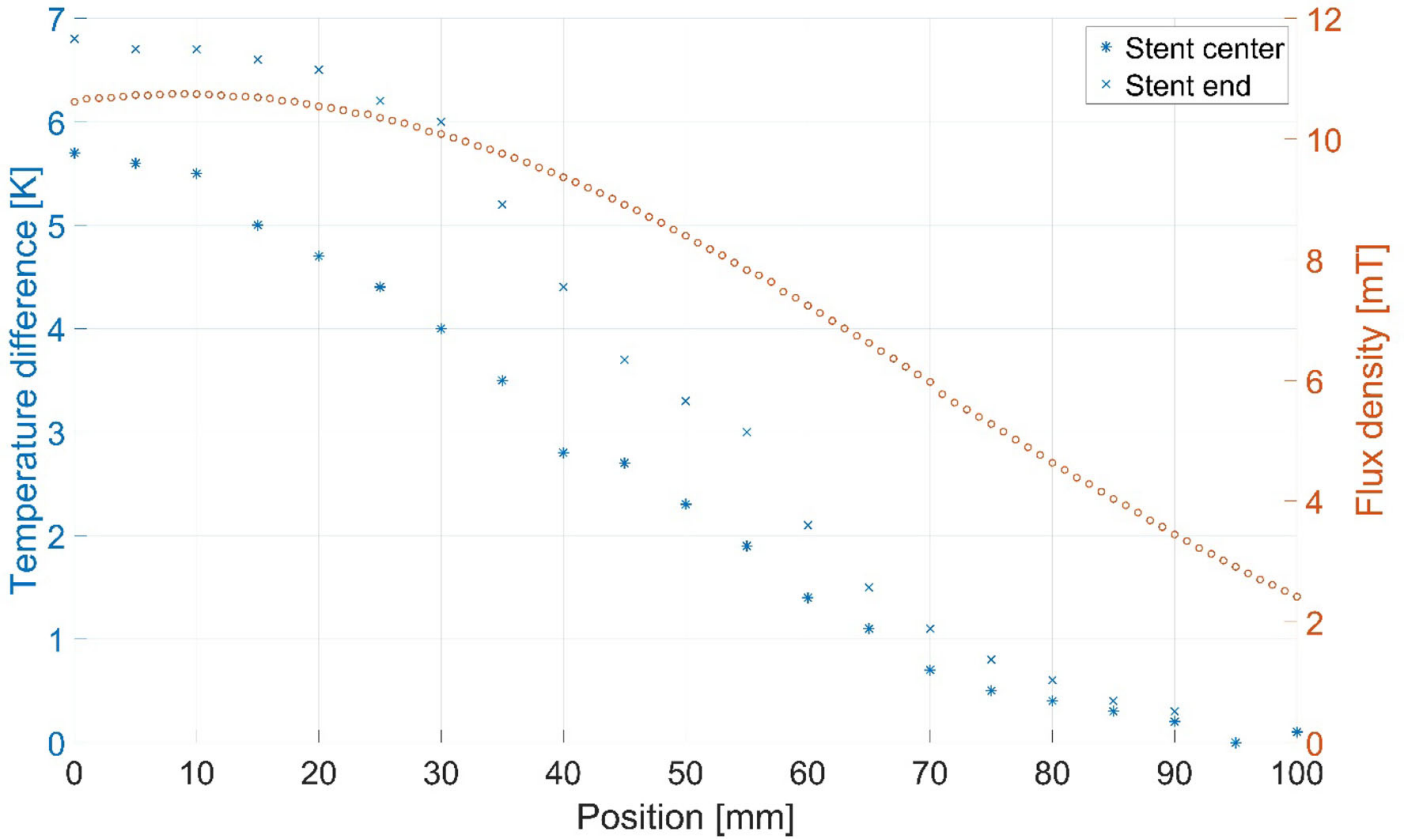
Variation in the stent position (Boston Scientific/Wallstent-Uni Endoprothesis) and measured temperature differences after 60 s of MPI scan at each location. In addition, the induced voltage in a pickup coil was measured and the magnetic flux density calculated to estimate the field distribution in x-direction. At the last two data points, the temperature measurement results are identical

The measured temperature differences showed a maximum (stent center 5.7 K, stent end 6.8 K) at the isocenter, the minimum at 95 mm from the isocenter (0 K), and were continuously decreasing with increasing distance from the isocenter (Fig. 5). The decrease in the heating had a nearly similar descent as the magnetic flux density with a linear reduction in the temperature values for 10 to 70 mm distance. The values measured at the stent end were slightly higher than the ones which were measured at the stent center for most of the positions.

## Discussion

This work includes four key messages which we assume to be important for the perspective of clinical MPI: First, stents with large diameters can cause heating which is dangerous for in vivo usage. Second, the drive field strength influences the heating of stents, but the selection field gradient does not affect the extent of heating for the given stents. Third, the profile of the magnetic field in the MPI scanner correlates with the intensity of stent heating. Fourth, the stent design influences the heating distribution of the stents.

The heating of metallic objects seems to be caused by two phenomena: the re-magnetization losses of magnetic domains and the induction of eddy currents. The latter is indicated to have a superior effect on heating of the stents under investigation. This is confirmed in this work by the lack of influence of the selection field gradients’ variation in heating. In principle, the static selection field could suppress re-magnetization of ferromagnetic domains caused by the oscillating drive field and thus influence the heating behavior of metallic objects.

In previous work, a correlation between stent diameter and measured temperature increase in oscillating magnetic fields was shown [16, 17, 19]. This relation seems to be based on the stent being a closed conductor loop. Here, the heating of re-dilatable Babystents in MPI was strictly reduced when the conductor loop was opened [16]. With respect to the biological effects of heating, it must be acknowledged that a temperature increase of 6 K can inhibit the proliferation of vascular smooth muscle cells [20] and also seems to be sufficient for stent-based tumor treatment [21]. As the blood flow is expected to have a cooling effect of more than 50% [22], only the tested stents with diameters of 16 mm or more caused heating, which is expected to be harmful for humans. Especially, the stents with diameters of 20 mm and 31 mm showed temperature differences which are potentially life threatening, as a burn of the (aortic) vessel wall can cause severe bleeding.

In addition, the stent design influences the heating and its distribution on the stent surface. The helical design, which caused heating maxima at the stent ends, seems to act as an electromagnetic coil. The ends two loops are partly touching each other, and thus, a high amount of eddy currents seems to be induced there. This is in accordance with observations made in MRI [23]. The open cell and closed cell design caused heating with the hot spots at the middle section of the stents. Here, it is assumed that the energy input is homogenous, with a slight heat loss at the stents ends. This goes along with previously observed distribution of stent heating after MPI scans [16, 17].

As human-scale systems may use smaller drive field strengths to avoid peripheral nerve stimulation and different gradient strength caused by power limitations, the variation in the MPI parameters was investigated. The reduction in the drive field caused a decrease in the temperature difference, which is in accordance with Faraday’s law of induction, if the dominant heating source is of inductive nature. According to this principle, the drive field strength should contribute quadratically to the heating. Due to the small amount of measurement points, it remains open, if the observed relation in this work is linear or quadratic. Nevertheless, based on these data, an increase in the drive field strength should only be considered with caution.

With respect to the described temperature increases, the question arises if stents outside the FOV can also be heated. Here, a continuous, mainly linear loss of temperature with increasing distance from the scanner’s isocenter was observed, which was also related to the scanner’s magnetic field topology. This is in accordance with the observed relation of the drive field strength and the stent heating. The reduction in the magnetic field is mainly caused by the geometric extend of the field generator itself. Thus, it can be assumed that a thoracic aortic stent, e.g., is not warmed up by the magnetic fields of a human brain scanner during cerebral imaging. We suggest taking the individual field survey of each scanner into account to estimate the stent heating effects outside the FOV.

As the increase in stent temperature has its maximum in the first two minutes of an MPI scan [16], the amount of heating in a clinical imaging scenario with shorter imaging sequences is expected to be less in comparison with the results of this study.

In this work, there are following limitations to acknowledge: It is an in vitro worst-case scenario study with static conditions and air as surrounding medium. Thus, to estimate the cooling effect of the blood flow, further studies are necessary. Furthermore, only one exemplary stent model of each stent design group was imaged once with thermography to identify the hot spot of the stents for the representative placement of the fiber-optic thermometers. Minor deviations due to slightly different design aspects may not have been noted with this approach. In general, the differentiation into three design groups may limit the detection of minor design aspects. Therefore, we propose a systematic design study, which focusses on the effects of the stent design in more detail. In addition, the number of stents and stent grafts which were evaluated in this work is limited. Due to the wide range of different models, each stent type should be reevaluated in vitro before in vivo usage. In a previous study with twenty-one different stents made from four different materials, we could not find a correlation between the heating effect and the type of material, while the diameter and the stent design had shown a major influence on the heating [17]. Since the last two factors could be confirmed, the correlation between material and heating is not addressed in this work, as mainly nitinol stents were investigated. Furthermore, the variation in the scan parameters is limited in its range in this study. Carrying out the measurements with more measurement points might reveal so far hidden relations. In addition, the heating of overlapping stents should be tested in the future. With the given perspective of clinical MPI, regulatory and legislative aspects of stent imaging must be kept in mind by the device manufacturers.

## Conclusion

This study demonstrates that stents with large diameters may show potentially harmful temperature increases in MPI. Besides stent design and diameter, the drive field strength, and the position of the stent in relation to the FOV are further influencing factors, whereas the selection field gradient had no effect. Taken together, these findings are the basis for further work to develop protocols for safe clinical imaging with MPI and allow for new large stent designs compatible with MPI by avoiding heating effects. Furthermore, this study illustrates the need for safety assessments of metallic stents by the manufacturers before MPI usage.
